# Co-infection with Drug-Susceptible and Reactivated Latent Multidrug-Resistant *Mycobacterium tuberculosis*

**DOI:** 10.3201/eid2111.150683

**Published:** 2015-11

**Authors:** Laura Pérez-Lago, Miguel Martínez Lirola, Yurena Navarro, Marta Herranz, María Jesús Ruiz-Serrano, Emilio Bouza, Darío García-de-Viedma

**Affiliations:** Gregorio Marañón University General Hospital, Madrid, Spain (L. Pérez-Lago, Y. Navarro, M. Herranz, M.J. Ruiz-Serrano, E. Bouza, D. García-de-Viedma);; Gregorio Marañón Health Research Institute, Madrid (L. Pérez-Lago, Y. Navarro, M. Herranz, M.J. Ruiz-Serrano, E. Bouza, D. García-de-Viedma);; Centro de Investigación Biomédica en Red en Enfermedades Respiratorias, Madrid (L. Pérez-Lago, Y. Navarro, M. Herranz, M.J. Ruiz-Serrano, E. Bouza, D. García-de-Viedma);; Torrecárdenas Hospital, Almería, Spain (M. Martinez Lirola);; Complutense University of Madrid, Spain (E. Bouza);; Moncloa International Campus of Excellence, Complutense University-Politécnica University, Madrid (Y. Navarro, D. García-de-Viedma)

**Keywords:** Mycobacterium, tuberculosis, mixed infection, co-infection, MDR, reactivation, recent transmission, genotype, Romania, Spain, antimicrobial resistance, bacteria, tuberculosis and other mycobacteria, TB

**To the Editor:** Genotyping of *Mycobacterium tuberculosis* (MTB) has identified mixed infections involving >1 MTB strain (*1*–*4*), which are clinically relevant when different susceptibility patterns are involved (*4*–*7*). We describe a tuberculosis (TB) case-patient with mixed infection in an area of moderate incidence. In a low-resistance setting (monoresistance 4.6%; multiresistance 1.7%), 1 of the strains was drug susceptible and the other was multidrug-resistant (MDR). Molecular fingerprinting and epidemiologic research revealed that the infection corresponded to a recent infection by a susceptible strain and reactivation of an MDR TB strain. The patient was an HIV-negative woman, 47 years of age, who had immigrated to Spain from Romania and had been living in Almería for >3 years. TB was diagnosed in May 2014; she had experienced symptoms for 2 months. Her diagnosis was confirmed 3 years after being studied in Almería as a close contact of her husband, also from Romania, who had tested positive for MDR TB (resistant to rifampin and isoniazid). When she was observed in the contact trace, she tested positive for purified protein derivative, had been vaccinated against the *M. bovis* bacillus Calmette-Guérin strain, and had no radiologic findings or clinical symptoms. Based on the susceptibility profile of her husband, prophylaxis was not prescribed. Her husband adhered to anti-TB treatment for 20 months; all microbiological control test results had been negative since 2 months after starting therapy.

Because her infection was thought to originate from previous contact with an MDR TB case-patient, we assessed her sputum samples for resistance using GenoType-MTBDR-plus (Hain-Lifescience, Nehren, Rhineland-Paltinate, Germany). The test showed hybridization with the same mutant probes (*rpo*B-MUT3; *kat*G-MUT1) as those of her husband, the assumed index MDR TB case-patient (Figurepanel A). The pattern was indeterminate because the hybridization for the mutant probes was faint, and intense hybridization was observed for all the *wt* probes (Figure panel A). Results suggested the simultaneous presence of an MDR strain and a susceptible strain in a respiratory specimen. The presence of the MDR TB strain was confirmed by the phenotypic antibiogram in a BBL MGIT mycobacteria growth indicator tube (Becton Dickinson, Franklin Lakes, NJ, USA) after the isolate had been cultured. GeneXpert (Cepheid, Sunnyvale, CA, USA) was used to analyze 2 respiratory specimens. Results indicated susceptibility to rifampin, revealing the limitations of this test: the use of probes targeting the *wt* sequences failed to detect resistant strains that coexist with a susceptible strain (*7*).

**Figure:**
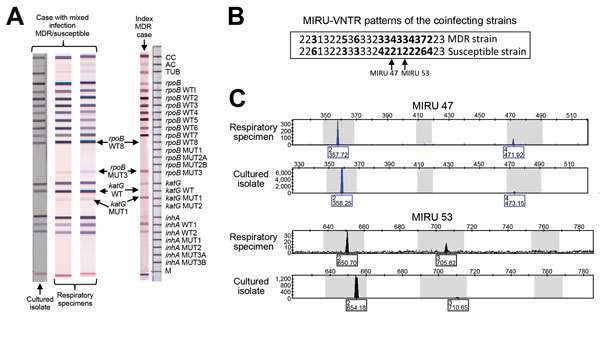
Identification of co-infection with drug-susceptible and reactivated latent multidrug-resistant Mycobacterium tuberculosis (MDR TB). A) Genotype of multidrug-resistant tuberculosis (MDR TB) and results for the MDR TB index patient and for 2 respiratory specimens and 1 cultured isolate from the case-patient who had a mixed MDR/susceptible infection. The same indeterminate intense-wt/faint mutant pattern test was repeated by using another 2 specimens. B) Mycobacterial interspersed repetitive units–variable number tandem repeat (MIRU-VNTR) types for the 2 strains involved in the co-infection. The results from the loci in which the analysis yielded double alleles are in bold text. Values for MIRU47 and 53 are indicated. C) Selection of 2 electropherograms representative of 2 (MIRU47 and 53) of the 12 loci with double alleles caused by mixed infection. Data that were obtained from direct analysis of a respiratory specimen or from a cultured isolate are shown.

To ascertain the likelihood of 2 co-infecting strains, we analyzed the specimen and the cultured isolate by mycobacterial interspersed repetitive unit–variable number tandem repeat (MIRU-VNTR), which is highly sensitive for detecting complex infections (*8*,*9*). Double alleles were found at 12 loci (Figure, panel B), confirming co-infection.

Peaks in the electropherograms suggested that 1 of the 2 strains was under-represented and its proportion was lower in the cultured isolates (Figure, panel C), indicating that culturing diminished its representation of the minority strain. This finding was consistent with the inability of the GenoType test to detect the MDR strain when applied to the cultured isolate (Figure, panel A). We also detected lower fitness for the MDR strain compared to the susceptible strain (p<0.01) (Technical Appendix Table).

Proportions of the resistant and susceptible strains were determined by plating on Middlebrook 7H11 +/− isoniazid (0.4 μL/mL) and counting single colonies. Because 2% of the colonies were of the resistant strain, we separated the strains and concluded: 1) the strain cultured in the presence of an antimicrobial drug coincided in the population exclusively with the MDR strain from the husband (as shown by MIRU-VNTR); and 2) the co-infecting MDR strain was a minority strain that was under-represented in the cultured isolate.

To analyze the origin of the susceptible strain, we investigated its MIRU-VNTR type in the population-based molecular epidemiology survey (*10*) and found another 4 cases (from 2008, 2011 [2 cases], and 2014). Three case-patients had emigrated from Romania, and all 5 case-patients lived in the same area of Almeria. These data indicated that the susceptible strain was circulating in the geographic/epidemiological context of the current case-patient before and when she tested positive for that strain; therefore, she likely acquired the susceptible strain through recent transmission.

The presence of susceptible and resistant strains in a patient should be considered even in moderate incidence settings and where resistance rates are not high. Underdetection of these cases could lead to misinterpretation when MDR became apparent after treatment of susceptible strains. Diagnostic laboratories could easily screen for mixed infections by applying MIRU-VNTR. However, only by integrating clonal analysis, refined molecular typing, and epidemiologic data from universal genotyping programs can we clarify the reasons underlying complex MTB infections. For this case-patient, a recent infection with a susceptible strain coincided with or could have triggered reactivation of a latent infection involving an MDR strain acquired through close contact years previously. We emphasize the alteration of the true clonal complexity of an infection induced by culturing specimens and that some commercial tests do not identify complex MTB infections. These findings are particularly relevant when the infection involves resistant strains such as those found in this case-patient.

Technical Appendix In vitro fitness of the multidrug-resistant and susceptible *Mycobacterium* *tuberculosis* strains involved in the mixed infection.
