# Supplementary material for: Co-infection with Drug-Susceptible and Reactivated Latent Multidrug-Resistant Mycobacterium tuberculosis
Source: Emerg Infect Dis. 2015 Nov;21(11):2098–100. doi: 10.3201/eid2111.150683 (PMC4622252; doi:10.3201/eid2111.150683)
Supplement: Technical Appendix — In vitro fitness of the multidrug-resistant and susceptible Mycobacterium tuberculosis strains involved in the mixed infection. [file 15-0683-Techapp-s1.pdf]

Article DOI: http://dx.doi.org/10.3201/eid2111.150683

# Co-Infection with Drug-Susceptible and Reactivated Latent Multidrug-Resistant *Mycobacterium tuberculosis*

## Technical Appendix

**Technical Appendix Table.** Fitness analysis, assayed in vitro*, for 2 co-infecting strains of drug-susceptible and reactivated latent multidrug-resistant *Mycobacterium tuberculosis*†

| Strains | Lag phase (hours to positive threshold [75 GU]) | Growth rate (hours for the 3000–4000 GU increase) |
|---|---|---|
| Susceptible strain | 220.17 ± 14.85 | 28 ± 1.13 |
| MDR strain | 289.67 ± 6.12 | 46.4 ± 2.26 |

*MGIT960 culture system, Becton Dickinson, Franklin Lakes, NJ, USA.
†Mean ± SD values were calculated for data from 3 independent experiments. GU, growth units.
